# Complete Genome Sequence of Mayaro Virus Imported from the Amazon Basin to São Paulo State, Brazil

**DOI:** 10.1128/genomeA.01341-15

**Published:** 2015-11-25

**Authors:** Mânlio Tasso Oliveira Mota, Danila Vedovello, Cassia Estofolete, Camila Dantas Malossi, João Pessoa Araújo, Maurício Lacerda Nogueira

**Affiliations:** a School of Medicine of São José do Rio Preto (FAMERP), São José do Rio Preto, SP, Brazil; b Universidade Estadual Paulista (Unesp), Botucatu, SP, Brazil

## Abstract

Mayaro (MAYV) is a neglected arbovirus from the tropical Americas. Here, we report the complete genome of an MAYV isolate from a patient returning from the Amazon basin and complaining of arthralgia, high fever, and headache, who was attended at an emergency service of São José do Rio Preto, São Paulo state, Brazil.

## GENOME ANNOUNCEMENT

Mayaro virus (MAYV) is an enveloped, positive-sense, single-stranded RNA arbovirus from the tropical Americas. It belongs to the genus *Alphavirus*, family *Togaviridae*. *Haemagogus* mosquitoes transmit the virus to primates and birds and sometimes to humans. MAYV has two genotypes, L, which circulates only in Pará state in the north region of Brazil, and D, which circulates more broadly in many countries of the tropical Americas and Caribe (1). MAYV causes a mild-to-severe Dengue-like febrile syndrome, very similar to that caused by Chikungunya (CHIKV), characterized by fever, headache, rash, malaise, myalgia, arthralgia and, sometimes, arthritis, which can be very debilitating and can persist for months (1).

The virus has been detected in several countries; however, it remains a neglected disease due to the inadequate surveillance in areas where it is endemic, and the generic nature of clinical manifestations that result in misdiagnosis with other viral fevers, mainly Dengue (2).

Here is reported the complete genome sequence of an MAYV strain isolated from a patient complaining of joint pain, high fever, and headache, attended at the emergency service of the Hospital de Base (HB) of São José do Rio Preto, São Paulo state, Brazil. The patient reported a work trip to the city of Portal in the interior of Pará state (Amazon basin) 40 days prior to his admittance. During this travel, the patient visited different populations, including riverside communities, and returned without symptoms to São Paulo state.

MAYV was first detected by reverse transcription (RT)-PCR, and then isolated in Vero E6 cells. Next-generation sequencing obtained a sequence of 11.438 nucleotides (nt), named BR/SJRP/LPV01/2014 and representing 99% of the genome. The genome has two coding regions, the first is the nonstructural polyprotein (nsP), covering the positions 87 to 7417 and with 2,442 amino acids (aa) of length. It codes four nonstructural proteins: nsP1, nsp2 (protease), nsP3, and nsp4 (RNA polymerase RNA dependent). The second is the structural polyprotein, between the positions 7442 to 11172, which is 1,242 aa in length and codes the capsid and envelop proteins (1–3) and the 6-k peptide. Separating these two coding regions are an internal ribosome entry site (IRES), between the position 7416 and 7441 nt. A subgenomic promoter between the position 7395 and 7418 nt precedes the IRES. There is an opal stop codon over the nsP, at position 1242. The 5′ untranslated region (UTR) has 86 nt and the 3′ UTR has 267 nt.

The phylogenetic reconstruction shows the isolated strain grouping within the L clade that occurs only in the Pará state and highlights the role of the high mobility of the population in the dispersion of the virus. This virus has great urbanization potential (3), following the steps of the CHIKV, which was originally limited to Africa but rapidly spread to Asian and Ocean Pacific countries, causing explosive outbreaks (3).

Awareness of health personnel about neglected viruses and continuous surveillance are important to detect circulation of these viruses outside their regions of endemicity.

### Nucleotide sequence accession number.

This genome has been deposited in GenBank under the accession no. KT818520. The version described in this paper is the first version.
